# Development of the Japanese Version of the Infertility Stigma Scale: Examination of Its Reliability and Validity

**DOI:** 10.3390/healthcare10030505

**Published:** 2022-03-09

**Authors:** Rie Yokota, Hiroko Okada, Tsuyoshi Okuhara, Eiko Goto, Emi Furukawa, Ritsuko Shirabe, Keiko Sakakibara, Takahiro Kiuchi

**Affiliations:** 1 Department of Health Communication, Graduate School of Medicine, The University of Tokyo, Tokyo 113-8655, Japan; efurukawa-tho@umin.ac.jp (E.F.); rshirabe-tky@umin.ac.jp (R.S.); 2 Department of Health Communication, School of Public Health, The University of Tokyo, Tokyo 113-8655, Japan; okadahiroko-tky@umin.ac.jp (H.O.); okuhara-ctr@umin.ac.jp (T.O.); gotoue-tky@umin.ac.jp (E.G.); tak-kiuchi@umin.ac.jp (T.K.); 3 Department of Social Psychology, Faculty of Sociology, Toyo University, Tokyo 112-8606, Japan; sakakibara@toyo.jp

**Keywords:** infertility, stigma, women, infertility stigma scale, reliability, validity, health communication

## Abstract

The stigma of infertility negatively affects health, resulting in mental distress and poor quality of life. An appropriate scale is essential to examine the stigma experienced by infertile women and provide adequate interventions. Therefore, we developed a Japanese version of the Infertility Stigma Scale (ISS). After examining the content validity of this scale, we conducted an online survey of women undergoing fertility treatment to test the scale’s structural validity, internal consistency, intra-rater reliability, known-groups validity, convergent validity, and discriminant validity. A total of 254 participants were included in the analysis. The results of confirmatory factor analysis of four factors based on the original scale revealed the optimal fit. Cronbach’s alpha was 0.95 for the total score. Concerning test–retest analysis, the total score of the ISS and subscale had a high Spearman correlation coefficient (*ρ* = 0.61–0.88, *p* < 0.001). For convergent validity, the association between the ISS and social support, self-esteem, and family functioning was significantly negatively correlated. The results of the multitrait scaling analysis scale showed that correlations of all items exceeded 0.40, and scaling errors (7/81, 8.6%) were few. The Japanese version of the ISS was confirmed to have acceptable reliability and validity.

## 1. Introduction

Infertility is defined as the failure to conceive after 12 months despite unprotected sexual intercourse [1]. In Japan, 18.2% of couples have undergone (or are currently undergoing) infertility testing or treatment: 1 in 5.5 of all couples [2]. In 2017, 56,617 babies were born in Japan through assisted reproductive technology, such as in vitro fertilization and intra-cytoplasmic sperm injection [3]. This is about 6.0% of the 946,065 total births in Japan [4]. In 2017, about 450,000 treatments were given, placing Japan among the top countries globally for administration of fertility treatments [5]. More than half (55%) of women undergoing advanced fertility treatment experience more than mild depression [5].

Despite the advances in fertility treatment, studies in other countries have indicated that the stigma of infertility has negative health consequences, including high psychological distress, poor quality of life, and social isolation [6,7,8,9,10,11,12]. Stigma refers to the process of labeling a group of individuals as having socially undesirable attributes, and these individuals are devalued by ‘a whole and usual person’ because of attributes and behaviors that are considered socially ‘deeply discrediting’ [13]. That is, stigma is when society negatively labels people who deviate from socially shared beliefs (i.e., a person should behave in a certain way at a certain time), so-called social norms and social expectations [14].

People in Japan who want a child but cannot conceive one may be viewed as ‘deviant’ and in a social minority [15,16]. There is a stigma against infertility in society, with discourse and images suggesting that ‘once married, a couple should have children’ and ‘a couple becomes a full-fledged couple when they have a child’. In many cases, women have internalized these values [17]. In fact, in a 2018 survey, 24.7% of women who had been married responded that couples are socially accepted only after having a child [18]. In a 2015 survey, 75.4% of never-married men and 67.4% of never-married women responded that they should have a child after marriage [2]. Thus, in Japan, there is a deep-rooted belief that the purpose of marriage is to bear a child, and a woman’s role is to produce children. Being infertile, therefore, presents many challenges for women in Japan [19], and many women who are infertile have internalized these traditional social norms [17]. Thus, the stigma regarding infertility needs to be examined in Japan.

Several studies have already examined the stigma of infertility by adapting commonly used tools such as the Adapted Stigma Consciousness Questionnaire, the Stigma of Fertility Problems Scale, and the Perceived Stigma Scale [6,10,20,21]. In addition, a female infertility stigma instrument is currently under development [22]. However, none of these scales have been examined for reliability and validity, and no Japanese tool exists to measure the stigma of infertility. The Infertility Stigma Scale was developed in China in 2015, and its reliability and validity were examined in China and Turkey [23,24]. This study examines the reliability and validity of the Japanese version of the Infertility Stigma Scale. This is because it is necessary to use reliable, valid, and effective measures of concepts in each culture and language in order to provide quality patient care [25]. In particular, since this study is focused on stigma, its reliability and validity may vary due to cultural bias. In other words, social norms and social expectations may vary from country to country. Therefore, it is necessary to examine the reliability and validity of the Japanese version of the Infertility Stigma Scale.

## 2. Materials and Methods

### 2.1. Scale Development

#### 2.1.1. The Infertility Stigma Scale

In this study, the Japanese version of the Infertility Stigma Scale (ISS) was targeted for development. The ISS was developed by Fu et al. in 2015 to assess the inner world of women living with infertility [23]: personal perception of stigma (perceived stigma) and feelings of loss of self-esteem, shame, and guilt (self-stigma). This scale consists of 27 items and four subscales: self-devaluation (7 items), social withdrawal (5 items), public stigma (9 items), and family stigma (6 items). Each item is rated on a 5-point Likert scale from totally disagree (1 point) to totally agree (5 points). The total score obtained from the scale ranges from 27 to 135. The Cronbach’s alpha coefficients of the original Chinese scale and the Turkish version [24] are 0.94 and 0.93, respectively. The Cronbach’s alpha coefficients of each subscale of the original scale were 0.86 for self-devaluation, 0.77 for social withdrawal, 0.92 for public stigma, and 0.84 for family stigma.

#### 2.1.2. Translation Process

After obtaining permission from the original authors of the ISS to translate and validate the instrument into Japanese, the Japanese version of the scale was generated following guidelines [26,27]. The translation process was conducted using a team approach with the aim of mitigating the subjective nature of translation and text-based translation evaluation [28,29]. The English version of this scale was translated into Japanese by one researcher who had no medical or clinical background and two researchers in the healthcare field. There was no significant difference between the three translations. After the forward translation, the reconciliation meeting was held with the three translators and one obstetrician/gynecologist (reviewer). Three translators plus a reviewer discussed their translations and wording, and also created translations that were more suitable for Japanese culture. Afterward, two experts in linguistics who did not have a medical background translated the scale back to English. After the back translation, the expert committee consisting of three doctors, two nurses, and three researchers was held. At the expert committee, three forward translations, a translation created after the reconciliation meeting, and two back translations, were discussed in terms of semantic, idiomatic, experimental, and conceptual equivalence. In particular, for items that cannot be assumed to relate to infertility, we referred to the Chinese version and added the phrase ‘because of infertility’. After these modifications were made, the members of the expert committee were asked to rate the Japanese translation of instructions, each item, response options, and each subheading on a 5-point scale from completely disagree to completely agree; 87.5–100% of the members agreed or completely agreed with each translation. Thus, the consensus was formed.

#### 2.1.3. Content Validity through Expert Panel

After the expert committee of the translation, the assessment of content validity was performed by the expert panel following guidelines and previous research [30,31,32,33,34,35]. An expert panel consisting of three doctors, two nurses, and three researchers provided opinions about the scale.

The word ‘stigma’ was used in some of the subscales, but might be difficult for the Japanese public to understand. The following sentence was thus added to the instructions: ‘The term ‘stigma’ appears in the questionnaire. This refers to the negative perception of an individual as being socially different from others.’ In addition, because reproductive medicine is available to common-law partners in Japan, the following sentence was added: ‘In addition, some of the items use the term “husband”. If you are in a common-law marriage, please read this as “partner” when you answer the questions.’ Item 5 is about family stigma: ‘My family, especially my mother-in-law, are always trying to make trouble for me.’ China has a high rate of coresidence with parents-in-law, but due to the low coresidence rate in Japan, the phrase ‘especially my mother-in-law’ was deleted.

After the expert panel discussion, panel members were asked to rate the essentiality, relevance, and clarity of each item. They also rated the comprehensiveness of each dimension and the entire instrument. Then, the content validity ratio (CVR), item-level-content validity index (I-CVI) for relevance, I-CVI for clarity, and modified kappa were calculated as a content validity indicator for each item to assess experts’ agreement on the need for the item. We also calculated the scale-level content validity index/average (S-CVI/Ave), scale-level content validity index/universal agreement (S-CVI/UA), and proportion of comprehensiveness as a content validity indicator for each dimension and the entire scale. The results of CVR, I-CVI, S-CVI/UA, S-CVI/Ave, and modified kappa are shown in Table A1 and Table A2. The CVR of each item exceeded 0.75, indicating that all items should remain [36]. I-CVI for relevance and clarity of each item was higher than 0.88, indicating that all items are relevant and clear [33]. Modified kappa of each item exceeded 0.87, indicating that interpretation criteria were excellent [30]. S-CVI/Ave and S-CVI/UA for relevance were 0.99 and 0.93, respectively. S-CVI/Ave and S-CVI/UA for clarity were 1.00 and 0.96, respectively. The proportions for agreement of comprehensiveness for each dimension and for the entire instrument were 87.5% and 100.0%, respectively. No items needed to be removed based on Polit’s approach [33].

After the evaluation by the expert panel, the Japanese version of the ISS was revised, back-translated, and confirmed as acceptable by the author of the original scale. We verified that the newly adapted scale did not differ significantly from the original authors’ intentions.

#### 2.1.4. Content Validity through Cognitive Interviews

As a pilot application and following empirical literature, the scale was administered to eight married women with no children [37,38]. Before the interviews, an interview guide was developed by referring to previous studies [32,39,40,41,42,43] (Table A3). The interviews were conducted online to prevent COVID-19 infection. The interviews were not conducted in groups but individually to manage the sensitive subject of stigma. The mean age of the participants was 37.4 years (SD 7.3 years). The educational background of the participants was 25.0% (2/8) with a two-year college degree and 75.0% (6/8) with a university degree or higher. In terms of employment status, four were full-time (50.0%), three were part-time (37.5%), and one was not working (12.5%). Because of the lack of epidemiological studies of infertile patients, the overall trend in Japan is not known [5], but when compared with the sociodemographic profile of the participants in the previous study [5], the mean age and the proportions of employment status were very similar, but the participants in this study had a higher educational background. All participants were interviewed after responding to the questionnaire. The mean interview time was about 30 min, and it took participants approximately 2 min 10 s to complete the ISS. All participants indicated that they felt no psychological stress when completing this scale. In the interview, participants were asked about general impressions of the scale, comprehensibility, relevance to infertile women’s experience, comprehensiveness of the scale, and appropriateness of response options. The percentage of agreement for comprehensibility and relevance is shown in Table A4. Regarding comprehensiveness, some participants found it difficult to understand the subheadings of ‘social withdrawal’ and ‘public stigma’. They were confused about the differences between social and public. We, therefore, changed the terms to ‘withdrawal in interpersonal relationships’ and ‘stigma in relation to people around them’ to reflect the intentions of the original author’s work. Apart from these changes, there were no major modifications, although some wording was altered. After interviews, the final draft was validated by the members of the expert panel.

### 2.2. Scale Validation

#### 2.2.1. Participants and Recruitment Procedure

To examine the validity and reliability of this scale, a web-based survey of women undergoing infertility treatment in Japan was conducted as a cross-sectional study in December 2021. We recruited women undergoing fertility treatment who were registered as monitors with a Japanese research company, and sent the survey questionnaire by e-mail through the research company.

The inclusion criteria were (1) women between 20 and 59 years old, (2) undergoing infertility treatment, (3) childless, (4) native Japanese speaking and living in Japan, (5) married (including common-law marriage), and (6) gave consent to participate in this study. Exclusion criteria were (1) women undergoing fertility testing, (2) having a background or experience in healthcare, and (3) diagnosed with mental illness.

When adapting a scale for different cultures, the sample size needs to be between five and ten times as large as the number of items in the scale [24,32]. Thus, the sample size was determined to be 254 women meeting the study criteria.

#### 2.2.2. Data Collection

Participants who consented to be enrolled in the study completed the questionnaire on the internet. To examine the Japanese version of the ISS, data were collected regarding three main variables: basic demographic information, infertility characteristics, and three validated measurements.

Basic demographic information included: age, duration of marriage, education, annual household income, occupation, and whether the couple lives alone or with their parents. Infertility characteristics included: duration of infertility, duration of infertility treatment, causes of infertility, and treatment for infertility.

The following three measures were used to examine convergent validity: the Multidimensional Scale of Perceived Social Support, the Rosenberg Self-esteem Scale, and the Family APGAR (as explained in greater detail in Section Examination of the Known-Groups, Convergent, and Discriminant Validity).

The Multidimensional Scale of Perceived Social Support was developed by Zimet (1988). This scale was designed to measure perceptions of social support adequacy from three sources: family, significant other, and friends [44,45]. This scale consists of 12 items, with each item graded on a 7-point rating scale from very strongly disagree (1 point) to very strongly agree (7 point). The mean of total and subscale scores ranged from 1 to 7, with higher scores indicating a higher perception of social support. The Japanese version of this scale for middle-aged and older people was developed by Iwasa et al. in 2007 [46]. Cronbach’s alpha coefficients of the Japanese version were calculated as 0.91 for the total score, 0.94 for family support, 0.88 for significant other, and 0.90 for friend support. The Cronbach’s alpha coefficient of the scale in this study was 0.93 for total score, 0.90 for family support, 0.88 for significant other, and 0.93 for friend support.

The Rosenberg Self-esteem Scale was developed by Rosenberg in 1965. It assesses global positive and negative attitudes towards the self [47]. This scale consists of 10 items, with each item of the scale graded on a 4-point Likert scale ranging from strongly disagree to strongly agree. The total scores range from 10 to 40, with higher scores indicating a higher level of self-esteem. The Japanese version of this scale was developed by Mimura and Griffiths in 2007 and validated by Uchida and Ueno in 2010 [48,49]. Cronbach’s alpha coefficients of the Japanese version were calculated as 0.81, and the Cronbach’s alpha for the scale developed in this present study was 0.90.

The Family APGAR was developed by Smilkstein in 1978 to assess subjective satisfaction with family functions [50,51]. This scale consists of five items covering family adaptation, partnership, growth, affection, and resolve. Each item of the scale is graded on a 3-point Likert scale ranging from hardly ever (0 point) to almost always (2 point). The total score ranges from 0 to 10, with higher scores indicating higher satisfaction with family functioning. The Japanese version of this scale was developed by Kokubu and Kamibeppu [52]. The Cronbach’s alpha coefficient of the Japanese version was 0.93 for adults, and the alpha value of the scale in this study was 0.89.

#### 2.2.3. Data Analysis

Data analysis was performed using R version 4.1.1. A *p*-value of < 0.05 was considered statistically significant. For the basic sociodemographic data and infertility characteristics data, the mean or percentage and standard deviation (SD) were calculated as the descriptive statistics.

##### Item Exclusion Criteria

Items were consideration for deletion based on examination of the ceiling/floor effect. Item-total and item-remainder correlations less than 0.4 or more than 0.85 were considered for deletion. An exploratory factor analysis was also conducted in which items were excluded if they had a factor loading of less than 0.4.

##### Examination of the Structural Validity

To assess the structural validity of the scale, exploratory factor analysis was conducted using the generalized least squares and varimax rotation after confirming factor numbers by a scree plot. In addition, based on confirmatory factor analysis, the factor structure of the ISS was examined in a theory-driven framework. Based on the original version of the ISS, a four-factor model was examined. Diagonal weighted least squares (DWLS) was used following Toyoda’s [53] recommendation. According to Toyoda, the results of Mardia’s kurtosis test for multivariate normality indicate that when a z value exceeds 3, the results may be inaccurate if the maximum likelihood estimation method is applied [53]. Thus, DWLS was chosen because the z value in this study exceeded 3 (z = 17.54) and included variables that were skewed distributions of continuous variables [53]. To evaluate the fit of the model, the chi-square statistic, chi-square statistic/degree of freedom (χ^2^/df), goodness of fit index (GFI), comparative fit index (CFI), Tucker–Lewis index (TLI), adjusted goodness of fit index (AGFI), normed fit index (NFI), root mean square error of approximation (RMSEA), and standardized root mean square residual (SRMR) were used [54]. A χ^2^/df value of 3 or lower is appropriate [55]. GFI, CFI, TLI, AGFI, and NFI of 0.95 or higher, and RMSEA and SRMR of 0.05 or lower refer to a good fit [24,56].

##### Examination of Reliability

Cronbach’s alpha coefficients, item-total and item-remainder score correlations were used to assess internal consistency. Cronbach’s alpha is suitable when it is ≥0.70 [57]. To assess the intra-rater reliability, retests were conducted three days after the initial survey. Following Cosmin’s criteria [32], retests were administered to 56 participants. After the survey, the test–retest reliability was estimated using the Spearman correlation coefficient, following the Turkish version [24].

##### Examination of the Known-Groups, Convergent, and Discriminant Validity

To assess known-groups validity, an independent Mann–Whitney U test was performed on duration of infertility. The scores were assumed to be higher when the duration of infertility was more than three years [8]. To examine convergent validity, Spearman’s correlation coefficient was calculated using the Multidimensional Scale of Perceived Social Support, the Rosenberg Self-esteem Scale, and the Family APGAR [46,48,52]. Previous studies have shown that social support, self-esteem, and family functioning are intimately related to stigma and similar scales were used in the original study [10,23,58,59]. A multitrait scaling analysis was performed to examine convergent and discriminant validity [60,61]. The convergent validity item was determined as a correlation ≥ 0.40 with the item and its own scale (adjusted for overlap). The discriminant validity item was sustained if the correlation of the item with its hypothesized scale (adjusted for overlap) was significantly higher than the correlation with any other scale. Convergent and discriminant validity were examined for each scale using the Spearman correlation coefficient. A scaling error was defined as when the item had a significantly lower correlation with its own scale (adjusted for overlap) than with another scale.

#### 2.2.4. Ethical Considerations

This study was conducted with the approval of the Ethics Committee of the University of Tokyo (approval number: 2021128NI), and informed consent was obtained from all participants.

## 3. Results

The participant characteristics are shown in Table 1. The mean (SD) age of participants was 35.9 (5.6) years. The mean (SD) of duration of infertility was 3.3 (2.9) years and the average (SD) duration of infertility treatment was 2.3 (2.4) years. More than two-thirds (69.6%) had a college degree or above, and 47.2% of the participants had undergone in vitro fertilization or microinsemination.

### 3.1. Item Score Distribution

The mean values and standard deviations of each item were calculated. The ceiling effect of one item and the floor effect of four items were observed. The mean (SD) of the total score of the ISS in this study was 73.6 (20.9).

### 3.2. Structural Validity: Exploratory Factor Analysis and Confirmatory Factor Analysis

An exploratory factor analysis was conducted after confirming that the questionnaire consists of four factors using a scree plot and without excluding any items. The factor structure revealed by using generalized least squares and varimax rotation is shown in Table 2. The factor loadings for the items comprising each factor were 0.44 or higher. The factor loadings for item 8 were larger for factor I than for factor II, which was different from the original factor structure. Item 14 had loadings of more than 0.4 on several factors, and the difference in loadings between the factor that items assigned to and the other factors was less than 0.1. The cumulative proportion of variance explained was 60%.

**Figure 1:**
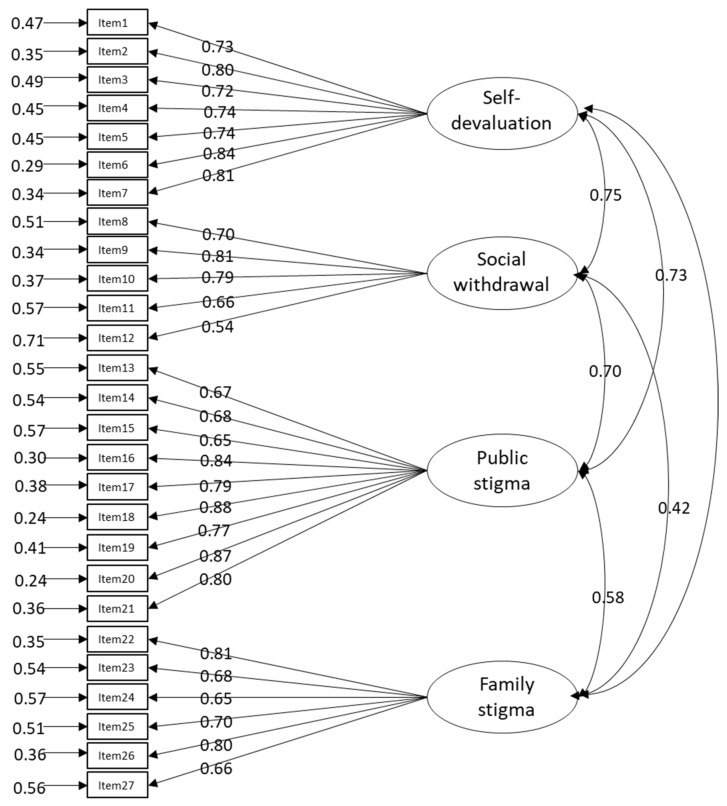
Standardized factor loadings for the ISS.

The results of confirmatory factor analysis using DWLS, categorized into 27 items with four factors, showed the optimal fit. The model fit indices were as follows: χ^2^ = 312.26, df = 318, χ^2^/df = 0.98, *p* = 0.58, CFI = 1.00, GFI = 0.98, TLI = 1.00, AGFI = 0.98, NFI = 0.98, RMSEA < 0.001 (90%CL, 0.00–0.02), SRMR = 0.06. The standardized factor loadings are shown in Figure 1.

### 3.3. Internal Consistency and Intra-Rater Reliability

The Cronbach’s alpha of the ISS and subscales was 0.95 for the total scale, 0.91 for self-devaluation, 0.83 for social withdrawal, 0.93 for public stigma, and 0.86 for family stigma (Table 3). The Cronbach’s alpha for each scale was 0.77–0.93 when each item was excluded (Table 4), lower than the values for each scale when any item was not excluded. The item-total correlations were 0.45–0.80 and item-remainder correlation 0.40–0.78 (Table 4). No items with weak or strong correlations were identified.

To examine inter-rater reliability, Spearman’s correlation coefficient was estimated for 56 participants who responded to a questionnaire administered three days after the initial administration (average age = 35.0 years). The Spearman correlation coefficient was 0.87 for the total score, 0.86 for self-devaluation, 0.88 for social withdrawal, 0.80 for public stigma and 0.61 for family stigma (Table 5).

### 3.4. Known-Groups Validity

A known-groups comparison was performed using the Mann–Whitney U test to examine clinical validity. The scale was feasibly able to discriminate between patients with different periods of infertility (Table 6). Patients with a longer duration of infertility (more than three years) tended to obtain a high score on the total score of the ISS (*p* = 0.004), self-devaluation (*p* = 0.006), social withdrawal (*p* = 0.044), public stigma (*p* = 0.006), and family stigma (*p* = 0.023).

### 3.5. Convergent Validity

To assess convergent validity, Spearman’s correlation coefficient was estimated between the ISS and the Multidimensional Scale of Perceived Social Support, the Rosenberg Self-esteem Scale, and the Family APGAR (Table 7). Higher total scores and scores on the subscales of the ISS were associated with lower scores on the Multidimensional Scale of Perceived Social Support, the Rosenberg Self-esteem Scale, and the Family APGAR.

### 3.6. Convergent and Discriminant Validity: Multitrait Scaling Analysis

To assess convergent and discriminant validity, multitrait analysis was conducted (Table 8). All of the items’ own correlations adjusted for overlap exceeded 0.40. Most of the items, except for ‘social withdrawal’, showed higher correlations with their own scale than with other scales. Scaling errors (7/81, 8.6%) were consistently low in all subscales.

## 4. Discussion

In this study, we surveyed women undergoing infertility treatment and developed a Japanese version of the ISS. The results showed the scale has high structural validity, internal consistency, intra-rater reliability, known-groups validity, convergent validity, and discriminant validity.

The mean age (SD) of the participants in the present study was 35.9 (5.6) years, similar to the average age of women in other studies of infertility in Japan [5]. In contrast, the mean age (SD) of the participants in the original study was 29.7 (4.4) years and 30.3 (5.6) years in the Turkish study [23,24]. Thus, the average age of the participants in our study was greater than that of the participants in the original and Turkish versions. Their educational level was also higher than the female participants in the other two studies. This may be because the level of education for both men and women has increased in Japan, which has raised the age of marriage and thus the age of childbearing [62].

Floor effects were observed in four items of stigma in the subscale of family stigma. This may be related to the low rate of cohabitation with parents in Japan. According to the survey of the National Institute of Population and Social Security Research, in Japan, the cohabitation rate of married daughters living with their parents in their twenties was 16.9%, for those in their thirties 14.5%, and for those in their forties, 17.3% [63]. It can be inferred that the parents’ traditional values are unlikely to influence the values of the couples. However, the mean scores (SD) of family stigma in the Chinese study and the Turkish version were 12.7 (5.2) and 9.4 (5.5), respectively [8,24]. Compared with the other studies, the mean score of 12.2 (5.2) for this study was not notably low.

The results of the exploratory factor analysis showed that as for the original study, the ISS consisted of four factors. All items had factor loadings of 0.44 or higher, exceeding the 0.4 criterion [24], and therefore no items needed to be excluded. However, Item 8 differed from the original factor structure. Item 14 also had loadings that exceeded 0.4 on several factors and the difference was less than 0.1 in loadings between the factor to which the item was assigned and the other factors. Thus, for these two items, the results were not consistent with the factor structure of the original version. This discrepancy may be attributed to the different configuration between the target population of the original version and that of this study. Further study should examine the measurement invariance between sample subgroups [64]. The cumulative proportion of variance explained by the original and Turkish versions was 58.2% and 57.9%, respectively, while the cumulative proportion of variance explained by the present study was 60%. In social sciences, an explained variance of 40% to 60% for multifactor patterns is adequate [24]. Thus, the cumulative proportion of variance explained in this study is sufficient. Although the factor structure differed from the original version for two items in exploratory factor analysis, the result of the confirmatory factor analysis of the four factors based on the original version revealed the optimal fit.

Cronbach’s alpha was 0.83 or more, higher than the original and Turkish versions [23,24]. Cronbach’s alpha when each item was excluded was 0.77–0.93, and there were no cases when the items were excluded when Cronbach’s alpha exceeded that of the complete scale. The item-total and the item-remainder correlations were 0.45–0.80 and 0.40–0.78, respectively. The item-total correlation of the Turkish version was 0.38–0.80 [24]. No items in this study were found to be inconsistent. Therefore, we decided not to exclude any items from this scale because there were no items that lacked internal consistency.

To measure time invariance, the test–retest method was performed. The Spearman correlation coefficient was used to examine the relationship between the two measurements. In the current study, the total score of the ISS and subscales for two measures exhibited a positive and high correlation (*ρ* = 0.61–0.88), and the correlation between the two measures was significant (*p* < 0.001).

When comparing the means of the groups with infertility duration of fewer than three years to those with infertility duration of more than three years, the scores of the four subscales were significantly higher in the group with infertility for more than three years. This indicates that the scores on this scale are linked to similar external criteria.

The association between the ISS and social support, self-esteem, and family functioning was significantly negatively correlated as in the original version. This indicates good convergent validity.

The results of the multitrait scaling analysis scale showed that (1) all the item-own correlations exceeded 0.40, and (2) scaling errors (7/81, 8.6%) were few. This suggests that, with some exceptions, convergent and discriminant validity of the ISS are good.

The mean total score on the ISS (SD) in this study was 73.6 (20.9). These scores were higher than infertile women undergoing in vitro fertilization (embryo transfer) in the Chinese study (mean 62.6, SD 21.6) and the participants in the Turkish study (mean 51.7, SD 23.0) [8,24]. This may be because secondary infertility was excluded in this study. The reasons for not including secondary infertility in this study were: (1) it is known that the level of stigma is higher for primary infertility than for secondary infertility [6]; (2) studies in developed countries typically use only primary infertility as an inclusion criterion [10]; and (3) it was difficult to consider secondary infertility as a societally deviant behavior in Japanese society because the total fertility rate in Japan is low at 1.36 in 2019 [65]. In addition, participants in the original and Turkish studies were recruited at medical institutions, while participants in the present study were recruited online. This may have reduced the social desirability bias and resulted in higher mean scores in the present study. The average score for stigma of mental illness was slightly higher than in China [66,67]. This shows that, despite the differences in inclusion criteria and recruitment, the level of stigma among our Japanese study participants was high. Being stigmatized is a reflection of Japan’s Gender Gap Index [68], which is low among developed countries (ranked 120th out of 156 countries), as it depends on the availability of social, economic, and political power [69]. Previous research has mentioned that: (1) in developed countries, voluntary childlessness is a more viable and legitimate option; (2) childless women are considered voluntarily childless; and (3) stigma of infertile women is high in developing countries [70]. Our study result is important because it reveals that stigma experienced by infertile women is high, even in developed countries.

There are several limitations to this study. First, the cognitive interviews in this study included participants who had not undergone fertility treatment. This was because the timing of the cognitive interviews coincided with the COVID-19 pandemic. To prevent infection, recruitment of participants at hospitals and clinics was abandoned, and snowball sampling was adopted. Regarding the representativeness of the sample, the participants in this study were similar to the participants in the previous study in terms of age and employment status, but differed in terms of education background [5]. Second, we did not test the reliability and validity of this scale on childless women who were not undergoing fertility treatment. The cognitive interviews suggested that women undergoing fertility treatment and childless women who have not undergone fertility treatment may have similar self-stigma and perceived stigma. As pointed out in the original version [23], there is the possibility of a scale that could be adapted for childless women not receiving fertility treatment in Japanese culture, but this has not been verified. Third, this scale did not include perceived stigma in medical situations. The participants who underwent infertility treatment indicated in the cognitive interviews that stigma may be perceived when they come into contact with pregnant women in the waiting rooms of hospitals and clinics. This point may be specifically related to the medical situation in Japan. Fourth, the Japanese version of the Multidimensional Scale of Perceived Social Support, which was used to verify the convergent validity of this study, was a scale that was examined for reliability and validity for middle-aged and older Japanese people, but it was not examined for reliability and validity for infertile women. Despite these limitations, this study is the first in the developed countries to test the reliability and validity of a measure of stigma among infertile women.

## 5. Conclusions

The Japanese version of the ISS was found to have acceptable reliability in terms of internal consistency, item-total and item-remainder correlation, and intra-rater reliability. Together with the results of the item analysis, it can be concluded that the Japanese version of the ISS has sufficient reliability to be used in intervention studies, and its content validity, construct validity, known-groups validity, and convergent and discriminant validity were acceptable. Although some items have been modified to fit the Japanese culture, the results of studies using this scale can be compared with Japanese efforts to reduce stigma among infertile women, and with those of other countries, helping to examine ways to reduce stigma.

## Figures and Tables

**Table 1:** Participant characteristics (*N* = 254).

	Mean	SD ^a^
Age (years)	35.93	5.55
Duration of marriage (years)	4.67	3.78
Duration of infertility (years)	3.32	2.88
Duration of infertility treatment (years)	2.27	2.44
	*N*	%
Education		
Less than high school	1	0.4
High school graduate	43	16.9
Vocational school graduate	33	13.0
Junior colleges or technical colleges	44	17.3
University graduate	125	49.2
Graduate school graduate	8	3.1
Annual household income		
Less than JPY 2,000,000	12	4.7
JPY 2,000,000 to JPY 4,000,000	38	15.0
JPY 4,000,000 to JPY 6,000,000	72	28.3
JPY 6,000,000 to JPY 8,000,000	59	23.2
JPY 8,000,000 to JPY 10,000,000	33	13.0
More than JPY 10,000,000	40	15.7
Occupation		
Office worker (regular employee)	87	34.3
Office worker (contract employee)	11	4.3
Public officer	10	3.9
Self-employed	13	5.1
Part-time worker	53	20.9
Housemaker	79	31.1
Unemployed	1	0.4
Living with parents		
Yes	15	5.9
No	239	94.1
Causes of infertility		
Male factor	15	5.9
Female factor	84	33.1
Both male and female factors	38	15.0
Unexplained factor	117	46.1
Treatment for infertility		
Timing therapy	74	29.1
Artificial insemination (AIH)	56	22.0
In vitro fertilization (IVF)	64	25.2
Microinsemination (ICSI)	56	22.0
Other	4	1.6

^a^ SD, standard deviation.

**Table 2:** Factor loading values of the ISS items (*N* = 254).

Item	Factor I Self-Devaluation	Factor II Social Withdrawal	Factor III Public Stigma	Factor IV Family Stigma
Item 1: Because of infertility, I feel that I have an unfortunate fate.	**0.65**	0.28	0.15	0.22
Item 2: Because of infertility, I feel that I am a failure to be a woman.	**0.69**	0.14	0.25	0.29
Item 3: Because of infertility, I feel like a burden to my family.	**0.59**	−0.01	0.34	0.25
Item 4: I feel inferior to others because of infertility.	**0.73**	0.27	0.20	0.08
Item 5: I am ashamed of being infertile.	**0.53**	0.29	0.36	0.08
Item 6: I look down on myself because of infertility.	**0.77**	0.14	0.31	0.19
Item 7: I feel useless at times because of infertility.	**0.76**	0.02	0.31	0.24
Item 8: I am more sensitive to pregnancy and child because I can’t get pregnant.	0.60	**0.44**	0.10	0.06
Item 9: I feel embarrassed when being asked something about the kids.	0.44	**0.59**	0.24	0.11
Item 10: I avoid getting close to people who don’t have fertility problem because of infertility,.	0.42	**0.53**	0.22	0.21
Item 11: I am unwilling to mention infertility.	0.16	**0.70**	0.27	0.08
Item 12: I try to conceal my condition from others.	0.12	**0.62**	0.24	-0.01
Item 13: It is common that people discriminate against infertile women.	0.27	0.20	**0.50**	0.22
Item 14: I dare not make new friends lest they find out that I have infertility.	0.12	0.48	**0.48**	0.24
Item 15: I worry that people may stay away from me when they find out I have infertility.	0.13	0.24	**0.57**	0.25
Item 16: I worry that people may look down on me when they find out I have infertility.	0.31	0.29	**0.71**	0.15
Item 17: I worry that people may laugh at me when they find out I have infertility.	0.22	0.22	**0.77**	0.15
Item 18: Because of infertility, I feel like a freak (an incomplete woman) in the eyes of others.	0.43	0.14	**0.69**	0.24
Item 19: I feel that people judge me behind my back because of infertility.	0.19	0.18	**0.74**	0.23
Item 20: I feel that people around me look down on me because of infertility.	0.34	0.19	**0.79**	0.18
Item 21: I feel that people view me differently because of infertility.	0.30	0.22	**0.67**	0.20
Item 22: Having infertility has spoiled my life.	0.15	0.16	0.25	**0.69**
Item 23: I worry that the relationship with my husband would be worse.	0.24	0.02	0.02	**0.80**
Item 24: I am afraid my husband would divorce with me.	0.15	0.00	0.10	**0.77**
Item 25: I feel that my family does not take care for me as much as before because of infertility.	0.01	0.14	0.31	**0.65**
Item 26: Because of infertility, my family was always trying to make trouble for me.	0.14	0.16	0.31	**0.63**
Item 27: I am afraid my remarriage would be affected, once people know my situation.	0.25	0.01	0.17	**0.55**
Proportion of variance explained	0.18	0.10	0.19	0.13
Cumulative proportion of variance explained	0.60			
KMO value	0.93			
Bartlett’s test	chi-squared 4712.64, *p* < 0.001			

**Table 3:** Cronbach’s α of the ISS (*N* = 254).

	Item Number	Mean	SD	Cronbach’s α
Total (ISS)	27	73.62	20.87	0.95
Self-devaluation	7	22.79	6.82	0.91
Social withdrawal	5	16.74	4.77	0.83
Public stigma	9	21.89	8.50	0.93
Family stigma	6	12.20	5.21	0.86

**Table 4:** Internal consistency of the ISS (*N* = 254).

Item Number	Mean	SD	Item-Total Correlation ^a^	Item-Remainder Correlation ^a^	α
					if Item Deleted
Self-devaluation					
Item 1	3.26	1.14	0.65	0.62	0.90
Item 2	3.10	1.23	0.71	0.68	0.89
Item 3	3.13	1.25	0.64	0.61	0.90
Item 4	3.84	1.14	0.66	0.63	0.90
Item 5	2.92	1.19	0.66	0.62	0.90
Item 6	3.35	1.20	0.73	0.70	0.89
Item 7	3.18	1.28	0.72	0.68	0.89
Social withdrawal					
Item 8	3.94	1.10	0.58	0.54	0.81
Item 9	3.74	1.18	0.68	0.64	0.77
Item 10	2.87	1.34	0.68	0.64	0.80
Item 11	3.05	1.26	0.56	0.51	0.78
Item 12	3.14	1.28	0.45	0.40	0.82
Public stigma					
Item 13	2.87	1.17	0.63	0.60	0.93
Item 14	2.21	1.10	0.65	0.62	0.93
Item 15	2.07	1.07	0.65	0.61	0.93
Item 16	2.60	1.18	0.77	0.74	0.92
Item 17	2.19	1.09	0.74	0.71	0.92
Item 18	2.67	1.29	0.80	0.78	0.92
Item 19	2.15	1.14	0.73	0.70	0.92
Item 20	2.49	1.25	0.80	0.78	0.92
Item 21	2.65	1.28	0.74	0.71	0.92
Family stigma					
Item22	2.06	1.09	0.58	0.55	0.84
Item 23	2.37	1.33	0.50	0.46	0.83
Item 24	1.80	1.03	0.48	0.45	0.83
Item 25	1.71	0.89	0.51	0.48	0.85
Item 26	2.01	1.11	0.60	0.56	0.84
Item 27	2.25	1.23	0.54	0.49	0.86

^a^ Item-total correlation and item-remainder correlation were calculated using the Spearman correlation coefficient.

**Table 5:** ISS test–retest analysis (*N* = 56).

Test–Retest Application	Spearman Correlation Coefficient
Total (ISS)	0.87 *
Self-devaluation	0.86 *
Social withdrawal	0.88 *
Public stigma	0.80 *
Family Stigma	0.61 *

* *p* < 0.001.

**Table 6:** Comparison between the ISS scores and duration of infertility (*N* = 254).

Duration of Infertility (years)	≤3 (*N* = 148)		>3 (*N* = 106)		U	*p*-Value
	Median	IQR ^a^	Median	IQR ^a^		
Total (ISS)	71.0	28.20	81.0	28.80	6180.0	0.004 **^b^
Self-devaluation	22.0	9.25	25.0	9.00	6247.5	0.006 **^b^
Social withdrawal	17.0	6.00	18.0	6.00	6683.5	0.044 *^b^
Public stigma	20.0	12.00	25.0	13.00	6247.5	0.006 **^b^
Family Stigma	11.0	8.00	12.5	9.00	6539.0	0.023 *^b^

* *p* < 0.05, ** *p* < 0.01; ^a^ Interquartile range; ^b^ Mann–Whitney U-test.

**Table 7:** Correlation of the ISS with the Multidimensional Scale of Perceived Social Support, the Rosenberg Self-esteem Scale, and the Family APGAR (*N* = 254).

		Total (ISS)	Self- Devaluation	Social Withdrawal	Public Stigma	Family Stigma
Multidimensional Scale of Perceived Social Support						
	Family	−0.36 **	−0.22 **	−0.22 **	−0.31 **	−0.48 **
	Significant other	−0.36 **	−0.23 **	−0.20 *	−0.30 **	−0.47 **
	Friends	−0.37 **	−0.28 **	−0.29 **	−0.33 **	−0.29 **
	Total score	−0.43 **	−0.29 **	−0.29 **	−0.38 **	−0.46 **
Rosenberg Self-esteem Scale		−0.51 **	−0.57 **	−0.33 **	−0.36 **	−0.36 **
Family APGAR		−0.32 **	−0.23 **	−0.12	−0.25 **	−0.44 **

* *p* < 0.01, ** *p* < 0.001.

**Table 8:** Multitrait scaling analysis between scale items on the ISS (*N* = 254).

	Item Number	Convergent Validity ^a^	Discriminant Validity ^b^	Scaling Errors
Self-devaluation	7	0.63–0.80	0.28–0.68	3 (14.3%)
Social withdrawal	5	0.52–0.70	0.19–0.58	4 (26.7%)
Public stigma	9	0.62–0.84	0.33–0.58	0
Family stigma	6	0.60–0.76	0.13–0.49	0

^a^ Item-own correlation scale corrected for overlap using Spearman correlation coefficient. ^b^ Item-other scale correlation using Spearman correlation coefficient.

## Data Availability

The data that support the findings of this study are available from the corresponding author, R.Y., upon reasonable request. The data are not publicly available due to ethical aspect.

## Appendix A

**Table A1:** Content validity ratio for the ISS through the expert panel (*N* = 8).

Item	Ne ^a^	CVR ^b^	Interpretation ^c^
Item 1	8	1.00	Remained
Item 2	8	1.00	Remained
Item 3	8	1.00	Remained
Item 4	8	1.00	Remained
Item 5	8	1.00	Remained
Item 6	8	1.00	Remained
Item 7	8	1.00	Remained
Item 8	8	1.00	Remained
Item 9	8	1.00	Remained
Item 10	7	0.75	Remained
Item 11	8	1.00	Remained
Item 12	8	1.00	Remained
Item 13	7	0.75	Remained
Item 14	8	1.00	Remained
Item 15	8	1.00	Remained
Item 16	8	1.00	Remained
Item 17	8	1.00	Remained
Item 18	7	0.75	Remained
Item 19	8	1.00	Remained
Item 20	8	1.00	Remained
Item 21	8	1.00	Remained
Item 22	7	0.75	Remained
Item 23	8	1.00	Remained
Item 24	8	1.00	Remained
Item 25	8	1.00	Remained
Item 26	7	0.75	Remained
Item 27	7	0.75	Remained

^a^ Ne: number of experts that evaluated essentiality of items. ^b^ CVR (content validity ratio) was computed using the formula: CVR = (Ne − *N*/2)/(*N*/2) with eight experts (where *N* = number of raters). ^c^ Interpretation criteria for CVR: Remained = CVR ≥ 0.75; Eliminated = CVR < 0.75.

## Appendix B

**Table A2:** Content validity index of item relevance and clarity, and modified kappa agreement for the ISS through the expert panel (*N* = 8).

Item	Relevance				Clarity				Interpretation ^d^
	Agreement	I-CVI ^a^	Pc ^b^	K ^c^	Agreement	I-CVI ^a^	Pc ^b^	K ^c^	
Item 1	8/8	1.00	<0.01	1.00	8/8	1.00	<0.01	1.00	Excellent
Item 2	8/8	1.00	<0.01	1.00	8/8	1.00	<0.01	1.00	Excellent
Item 3	8/8	1.00	<0.01	1.00	8/8	1.00	<0.01	1.00	Excellent
Item 4	8/8	1.00	<0.01	1.00	8/8	1.00	<0.01	1.00	Excellent
Item 5	8/8	1.00	<0.01	1.00	8/8	1.00	<0.01	1.00	Excellent
Item 6	8/8	1.00	<0.01	1.00	8/8	1.00	<0.01	1.00	Excellent
Item 7	8/8	1.00	<0.01	1.00	8/8	1.00	<0.01	1.00	Excellent
Item 8	8/8	1.00	<0.01	1.00	8/8	1.00	<0.01	1.00	Excellent
Item 9	8/8	1.00	<0.01	1.00	8/8	1.00	<0.01	1.00	Excellent
Item 10	8/8	1.00	<0.01	1.00	8/8	1.00	<0.01	1.00	Excellent
Item 11	8/8	1.00	<0.01	1.00	8/8	1.00	<0.01	1.00	Excellent
Item 12	8/8	1.00	<0.01	1.00	8/8	1.00	<0.01	1.00	Excellent
Item 13	8/8	1.00	<0.01	1.00	8/8	1.00	<0.01	1.00	Excellent
Item 14	7/8	0.88	0.03	0.87	8/8	1.00	<0.01	1.00	Excellent
Item 15	8/8	1.00	<0.01	1.00	8/8	1.00	<0.01	1.00	Excellent
Item 16	8/8	1.00	<0.01	1.00	8/8	1.00	<0.01	1.00	Excellent
Item 17	8/8	1.00	<0.01	1.00	8/8	1.00	<0.01	1.00	Excellent
Item 18	8/8	1.00	<0.01	1.00	8/8	1.00	<0.01	1.00	Excellent
Item 19	8/8	1.00	<0.01	1.00	8/8	1.00	<0.01	1.00	Excellent
Item 20	8/8	1.00	<0.01	1.00	8/8	1.00	<0.01	1.00	Excellent
Item 21	8/8	1.00	<0.01	1.00	8/8	1.00	<0.01	1.00	Excellent
Item 22	7/8	0.88	0.03	0.87	8/8	1.00	<0.01	1.00	Excellent
Item 23	8/8	1.00	<0.01	1.00	7/8	0.88	0.03	0.87	Excellent
Item 24	8/8	1.00	<0.01	1.00	8/8	1.00	<0.01	1.00	Excellent
Item 25	8/8	1.00	<0.01	1.00	8/8	1.00	<0.01	1.00	Excellent
Item 26	8/8	1.00	<0.01	1.00	8/8	1.00	<0.01	1.00	Excellent
Item 27	8/8	1.00	<0.01	1.00	8/8	1.00	<0.01	1.00	Excellent
S-CVI/Ave ^e^	0.99				1.00				
S-CVI/UA ^f^	0.93				0.96				

^a^ I-CVI (item-level content validity index) was computed using the formula: A/*N* where A = number of raters who agree that the item is relevant or clear and *N* = number of raters. ^b^ Pc (probability of a chance occurrence) was computed using the formula: Pc = [*N*!/A!(*N* − A)] 0.5^*N*^. ^c^ K (modified kappa) was computed using the formula: K = (I-CVI − Pc)/(1 − Pc). ^d^ Interpretation criteria for kappa: Fair = K of 0.40 to 0.59; Good = K of 0.60 to 0.74; Excellent = K > 0.74. ^e^ S-CVI/Ave: Scale-level content validity index/Average. ^f^ S-CVI/UA: Scale-level content validity index/Universal agreement.

## Appendix C

**Table A3:** Semistructured cognitive debriefing interview guide.

Topics	Probing Question
General impression of the scale	What were your feelings when you were answering the questionnaire? Did you experience any psychological distress while completing the questionnaire?
Comprehensibility	How comprehensible was the instructional text to you? How comprehensible was item X to you? How comprehensible were the response options to you? Are there any questions that would be better phrased in a more specific way?
Relevance	Does item X relate to the situation of infertile women? Are there any questions that are duplicated, repeated, or very similar?
Comprehensiveness	Is there anything missing from this questionnaire regarding the feeling that infertile women are socially different compared with others? To what extent did you feel that the content of this questionnaire covered the sense of infertile women that you just described? Is the grouping of questions appropriate?
Response options	Are the response options appropriate?
Suggestions for improvement	Do you have any suggestions on how to improve the questionnaire?

## Appendix D

**Table A4:** The percentage of agreement of comprehensibility and relevance through cognitive debriefing (*N* = 8, %).

Item	Comprehensibility	Relevance
Instruction	87.5	
Item 1	100.0	100.0
Item 2	100.0	100.0
Item 3	87.5	87.5
Item 4	100.0	100.0
Item 5	87.5	100.0
Item 6	100.0	100.0
Item 7	75.0	100.0
Item 8	100.0	100.0
Item 9	87.5	100.0
Item 10	100.0	100.0
Item 11	100.0	100.0
Item 12	100.0	100.0
Item 13	100.0	100.0
Item 14	87.5	100.0
Item 15	100.0	100.0
Item 16	100.0	100.0
Item 17	100.0	100.0
Item 18	87.5	100.0
Item 19	87.5	100.0
Item 20	100.0	100.0
Item 21	100.0	100.0
Item 22	75.0	100.0
Item 23	100.0	100.0
Item 24	100.0	100.0
Item 25	100.0	75.0
Item 26	100.0	100.0
Item 27	62.5	100.0
Response options	87.5	
